# Trimethoxystilbene Reduces Nuclear Factor Kappa B, Interleukin-6, and Tumor Necrosis Factor-*α* Levels in Rats with Pulmonary Artery Hypertension

**DOI:** 10.1155/2019/1219848

**Published:** 2019-11-26

**Authors:** Jie Shu, Wei Liu, Fei Han, Fanyan Luo

**Affiliations:** The Department of Cardiothoracic Surgery, Xiangya Hospital, Central South University, Changsha, Hunan Province, China

## Abstract

Pulmonary artery hypertension is a refractory disease that severely affects cardiopulmonary function, mainly resulting in irreversible pulmonary vascular remodeling. Current surgical treatment of this disease is not very effective and drug treatment is targeted at relieving symptoms, improving the quality of life of patients, and preventing disease progression. The purpose of this present study was to reveal the regulatory effects of trimethoxystilbene on the serum levels of nuclear factor kappa B, interleukin-6, and tumor necrosis factor-*α* in a rat model of pulmonary artery hypertension and to explore the possible underlying mechanisms. Healthy Sprague Dawley rats were randomly assigned to experimental groups and treated with monocrotaline to establish the model, and we found a significant difference in the expression levels of nuclear factor kappa B, interleukin-6, and tumor necrosis factor-*α* between the experimental and control groups. These results suggest that trimethoxystilbene significantly reduced the inflammatory factor levels in pulmonary hypertensive rats, providing us with new potential strategies for elucidating the mechanisms of action of trimethoxystilbene in the treatment of pulmonary artery hypertension.

## 1. Introduction

The main characteristics of pulmonary artery hypertension (PAH) are increasing pulmonary artery pressure and irreversible pulmonary vascular remodeling [1, 2] caused by pulmonary artery occlusion. In the pathogenesis of PAH, pulmonary vascular remodeling leads to abnormal trafficking between pulmonary and systemic circulation, resulting in continuous interventricular wall pressure and shear stress [3]. This process stimulates the secretion of a variety of inflammatory cells that infiltrate the remodeling pulmonary artery and release a large number of cytokines that play a significant role as chemokines, which induce adhesion and further aggravate the pressure within the ventricles [3]. These activities lead to ventricular wall thickening and the release of additional inflammatory factors in myocardial cells [3]. The immune response caused by inflammatory mechanisms that involve multiple serological factors is critical to the development of PAH, which is a malignant progressive disease with a poor prognosis and a high mortality [4, 5].

Currently, the study of PAH treatments mainly focuses on new vasodilators, such as prostacyclin analogs and 5-HT inhibitors. These medicines can relieve the symptoms and delay the progression of PAH, but they fail to achieve the goal of a cure, and new pharmacological interventions have focused on inhibiting a wide variety of proliferation factors and the secretion of inflammatory cytokines. Therefore, although the targets for the treatment of PAH are numerous, the suppression of inflammatory factors should be a new direction in the development of alternative treatment strategies. Resveratrol (Res) is a polyphenolic flavonoid compound present in a wide variety of natural edible and medicinal plants and is a plant toxin (phytoalexin) [6] that confers resistance against foreign invasion. The understanding of the pharmacological effects of Res was derived from a World Health Organization (WHO) epidemiological survey, i.e., the Monica Project, which revealed “the French Paradox.” The French Paradox is a phenomenon in which a high-fat diet is negatively correlated with the incidence of coronary heart disease, and additional studies have shown that the Res in red wine has a protective effect on the heart [7].

Subsequent studies confirmed that Res has various activities, such as antioxidant effects, cell cycle blockade, inhibitory effects against nuclear factor kappa B (NF-*κ*B), AP-1, cyclooxygenase- (COX-) 2, matrix metalloproteinases (MMPs), tumor necrosis factor- (TNF-) *α*, and interleukin- (IL-) 1*β* activities, and an estrogen-like effect [8, 9]. Trimethoxystilbene (TMS) is a derivative of 3,5,4-trihydroxy-trans-stilbene, also known as Res. We investigated TMS as a raw material in experimental research studies and were able to increase its lipid solubility using a methylation reaction-based synthesis process that introduced three methyl groups, which enhanced the ability of TMS to penetrate the cell membrane. This modification improved the bioavailability and enhanced the distribution of TMS to different organs and tissues in the body without inducing toxic reactions [10]. The present study aimed to explore the effects of TMS on PAH pathogenesis by determining differences in the relative transcription factor and cytokine expression levels in the lung tissue of a Sprague Dawley (SD) rat model of PAH after intervention with TMS treatment.

## 2. Materials and Methods

### 2.1. Experimental Animals

Thirty-six healthy SD rats provided by the Central South University Experiment Center which composed of an equal number of males and females weighing 200–250 g (certification: SCXK Hunan 2015-0003) were used in the study.

### 2.2. Experimental Drugs

The TMS used was a product of research by Central South University, School of Chemistry and Engineering, and it had a purity of 98.5%. TMS was prepared as a 120 mg/mL solution for treatments.

### 2.3. Experimental Methods

#### 2.3.1. Model Establishment and Animal Grouping

Thirty-six SD rats were randomly divided into three groups of 12 rats as follows. The model control (A) group was subcutaneously treated with dimethyl sulfoxide (Beyotime, Shanghai, China) at a dose of 30 mg/kg. The experimental model consisted of 24 rats subjected to adaptive breeding for 1 week and then subcutaneously injected with 30 mg/kg monocrotaline (Beyotime, Shanghai, China) for 7 consecutive days. Then, 45 days after the injection, we performed an ultrasound B-scan. After anesthesia with pentobarbital, the rats were fixed in the supine position, and the hair was removed from the chest. The ultrasound probe for small animals was placed on the left chest of the rats and connected to the ultrasonic test machine (VEVO3100, VISUALSONICS, Canada). We measured the right ventricular diastolic wall thickness, right ventricular systolic wall thickness, and ejection fraction. Then, myocardial hematoxylin and eosin (H&E) staining and terminal deoxynucleotidyl transferase (TdT) deoxyuridine 5′-triphosphate (dUTP) nick-end labeling (TUNEL) were conducted to confirm the successful establishment of the PAH model. Then, the PAH model animals were randomly divided into the model (B) and drug-treated (C) groups (*n* = 12) according to weight (kg) and were administered normal saline and TMS treatment, respectively, once daily.

*(1) Drug Administration and Animal Monitoring*. After the rat model was successfully established, the vehicle and test drug treatment dosages were calculated based on weight. Groups B and C were administered 5% normal saline solution and TMS, respectively, both at 120 mg/(kg/day) once daily, for 6 consecutive weeks, and the food intake, activities, fur color, and changes in urine and stool of the rats were observed daily. Furthermore, the rats were weighed every week.

#### 2.3.2. NF-*κ*B, IL-6, and TNF-*α* Detection in Myocardial Cells Using Immunohistochemistry with 3,3′-Diaminobenzidine (DAB)

The rats were sacrificed by cervical dislocation. We determined the expression of NF-*κ*B, IL-6, and TNF-*α* in prepared paraffin-embedded tissue samples from myocardial specimens of SD rats modeling PAH. We detected the expression levels of NF-*κ*B, IL-6, and TNF-*α* using immunohistochemistry with 3,3′-diaminobenzidine (DAB) as the chromogen. The presence of brown or brown-to-yellow granules in the cytoplasm of the positive reference slides and their absence in the cytoplasm of the negative reference slides in the same testing batch indicated that the experiment met the quality control requirement.

The myocardial cells in the tissue samples were observed at 400x magnification under a light microscope. Cells that showed the presence of brown or brown-to-yellow granules in the cytoplasm were considered positive. The observation view was randomly selected at the edge of the broken myocardial tissue on every slide. We observed and counted 200 cells in each field of view and counted the number of positively stained cells, which was used to determine NF-*κ*B, IL-6, and TNF-*α* expression.

After the rat myocardial tissue was obtained, total protein was extracted by RIPA lysis (Beyotime,Shanghai, China), and the protein concentration was measured by using a BCA protein assay kit (TaKaRa, Tokyo, Japan). Then, the protein sample was prepared and denatured at 100°C for 5 min. Next, 10% SDS-PAGE gels were prepared, and proteins were separated by electrophoresis. The proteins were then transferred to a PVDF membrane. Membranes were blocked in 5% STSA blocking solution formulated with TBST for 2 h. After blocking and washing, the following antibodies were incubated with the samples overnight at 4°C: rabbit antirat recombinant IL-6, rabbit polyclonal anti-IL-6 (Abcam, Cambridge, UK; dilution, 1 : 1000), rabbit polyclonal anti-NF-*κ*B p65 (Abcam, Cambridge, UK; dilution, 1 : 2000), rabbit polyclonal anti-TNF-*α* (Abcam, Cambridge, UK; dilution, 1 : 1500), and anti-*β*-actin (Abcam,Cambridge, UK; dilution, 1 : 5000). Next, the membrane was washed and incubated with goat antirabbit secondary antibody (Abcam,Cambridge, UK; dilution 1 : 2000). Finally, we used an enhanced chemiluminescence (ECL, Biovision, LA, USA) kit to observe protein bands in a ChemiDoc XRS Plus luminescent image analyzer (Bio-Rad, California, USA). *β*-actin was used as the internal control in the experiment, and therefore, the ratio of the integrated optical density of the targeted protein bands to that of *β*-actin in every group was determined as the final relative protein expression intensity. All experiments were replicated 3 times.

#### 2.3.3. IL-6 and TNF-*α* Detection in the Peripheral Blood of PAH Model Rats Using Enzyme-Linked Immunosorbent Assay (ELISA)

Six weeks after the PAH rat model was established, we randomly collected peripheral blood samples from the animals in every group. Then, enzyme-linked immunosorbent assay (ELISA) kits (Hengyuan Biotechnology Development Co., Ltd. Shanghai, China) were used to analyze the serum levels of biomarkers, IL-6 and TNF-*α*, strictly according to the manufacturer's instructions. Two blank controls were used for each specimen according to the instructions in the kit manual. Finally, we measured the absorbance (A) value at 450 nm using a microplate reader and calculated the concentration.

### 2.4. Statistical Analysis

We used the Statistical Package for the Social Sciences (SPSS) 20.0 statistical software to analyze all the data. Measurement data are presented as the mean ± standard deviation (*x* ± SD). Variables in skewed distributions were logarithmically transformed to a normal distribution before the analysis was performed. We used the one-way ANOVA with a correction test for multiple comparisons and LSD for comparisons between 2 variables. *P* < 0.05 indicated statistical significance.

## 3. Results

### 3.1. Confirmation of Model Establishment

**Figure 1:**
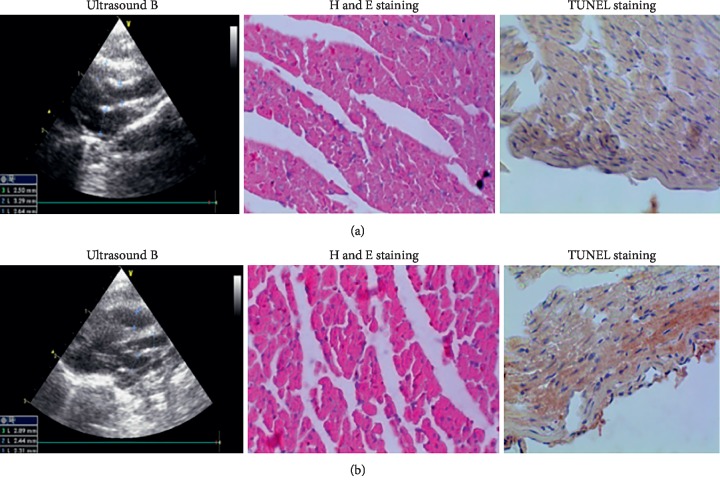
The results of the ultrasound B hematoxylin and eosin (H&E) staining and terminal deoxynucleotidyl transferase (TdT) deoxyuridine 5′-triphosphate (dUTP) nick-end labeling (TUNEL) staining of myocardial cells. Images were acquired with light microscopy (LM) under 40x magnification. (a) Control group. (b) Model group.

Ninety days after the animals in the experimental model groups were injected with monocrotaline, they manifested symptoms of heart failure, including dyspnea, the occurrence of pleural effusion and ascites, congestion and swelling of the liver, enlargement of the heart, and right ventricular wall hypertrophy. Ultrasonic examination revealed that the main changes in the experimental group were tricuspid regurgitation rate >3.4 m/s and pulmonary artery systolic pressure >50 mmHg. Other changes included an increase in the pulmonary valve regurgitation rate and a small decrease in the right ventricular ejection time. Furthermore, there was an increase in the right heart cavity diameter, abnormalities in the shape and motion of the interventricular septum, an increase in right ventricular wall thickness, and expansion of the main pulmonary artery. The H&E staining and TUNEL assay of the myocardial cells showed hypertrophy of myocardial cells with a disordered arrangement. The TUNEL assay showed an increase in the apoptosis of myocardial cells, which indicated the successful establishment of the SD rat model of PAH using monocrotaline. The results are shown in Table 1 and Figure 1.

### 3.2. NF-*κ*B, IL-6, and TNF-*α* Expression Detected in Myocardial Cells Using Immunohistochemistry (DAB Method)

**Figure 2:**
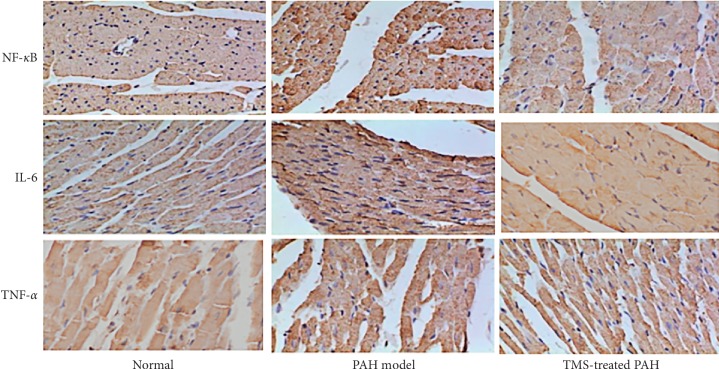
Expression levels of nuclear factor kappa B (NF-*κ*B), interleukin- (IL-) 6, and tumor necrosis factor- (TNF-) *α* in myocardial cells using immunohistochemistry (DAB method).

We observed and counted 200 cells in the rat tissue specimens and determined the number of positively stained cells, which was indicative of the positive expression rate of NF-*κ*B, IL-6, and TNF-*α* (Table 2). NF-*κ*B, IL-6, and TNF-*α* were moderately expressed in the myocardial cell nuclei of the control group, at an amount that was less than that in the myocardial cell nuclei of the model group, which showed high expression levels (Figure 2 and Table 2). However, these factors were only partially expressed in the TMS-treated group, indicating that their levels were significantly downregulated compared with those in the model control group (*P* < 0.01).

### 3.3. Expression Levels of NF-*κ*B, IL-6, and TNF-*α* in the Myocardial Cells of PAH Model Rats as Determined Using Western Blotting

**Figure 3:**
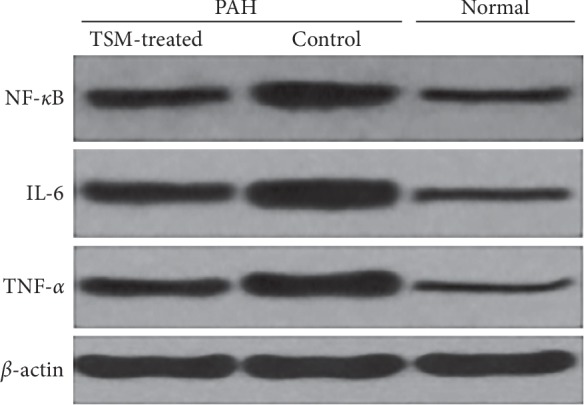
Protein expression of NF-*κ*B, IL-6, and TNF-*α* in the myocardial cells of the experimental PAH model rats as determined using western blotting.

We used western blot analysis to detect the protein expression of NF-*κ*B, IL-6, and TNF-*α* in the myocardial cells of the experimental PAH model rats. The results showed that the expression level of these factors in the myocardial cells of the PAH model SD rats was significantly higher than that of the normal control group rats, while the expression level of the TMS-treated group was significantly lower than that of the model control group (Figure 3 and Table 3). The blot exposure results were analyzed using Quantity-One gray scanning software, and the intensity is shown as the respective factor grayscale value/*β*-actin grayscale value.

### 3.4. Expression Levels of IL-6 and TNF-*α* in the Serum of PAH Model Rats as Determined Using ELISA

We used an ELISA to detect the protein expression levels of IL-6 and TNF-*α* in the serum of the PAH model SD rats. The results revealed that the expression level of these cytokines in the serum of rats in the PAH model group was significantly higher than that of rats in the normal control group, and the level of the TMS-treated group was significantly lower than that of the model control group (Table 4).

## 4. Discussion

PAH is a group of major pulmonary vascular diseases characterized by a progressive increase in pulmonary artery pressure [11]. An average pulmonary artery pressure >25 mmHg under resting conditions or >30 mmHg during movement with a corresponding pulmonary capillary wedge pressure (PCWP) < 15 mmHg constitutes a diagnosis of PAH. The main characteristic of PAH is a progressive increase in pulmonary artery resistance, which eventually leads to right-sided heart failure [12]. The premise for performing this specific research on PAH was to successfully establish an experimental model of pulmonary hypertension. According to reports in the literature, previously established PAH rat models include the monocrotaline-induced model, the hypoxia model, and the high pulmonary blood flow combined with monocrotaline induction model [6–8, 13].

Res is a natural and nontoxic substance, and a large number of studies have already confirmed its pharmacological effects, including the inhibition of cell DNA synthesis and oxidative stress-induced transcription factors and the induction of cell apoptosis and cell cycle arrest, leading to the inhibition and control of cell proliferation and blocking the formation of pannus. The antioxidant and antiplatelet aggregation effect of Res plays a protective role in the function of the cardiovascular system, liver, and kidney. In a recent study, we found that Res significantly reduced airway resistance in an airway hyperresponsive model established using ozone attack, which achieved a similar effect to that of the hormone treatment group [14]. Wang et al. [15] previously performed a pharmacokinetic experiment using Res in an animal model, and the results suggested that the maximum peak concentration (*C*_max_) in the blood of rats was 1.929 mg/L, the time to achieve *C*_max_ (*T*_max_) was 10 min, and the half-life (*t*_1/2_) was 11.5 min after a single gavage treatment with 10 mg/kg Res. These results indicate that Res has a relatively short *t*_1/2_.

To treat osteoarthritis, Elmali et al. [14] injected Res into the articular cavity, which was the only effective route for this treatment. However, this invasive administration routine limits the clinical use of this medication. Simoni et al. [16] modified the structure of Res and found that the 5-trimethoxy derivative showed high lipophilicity, which enhanced its bioavailability and inhibitory activity against cell proliferation by 2 folds. We modified the chemical structure of Res in this study and synthesized TMS with Res as the raw material using a methylation reaction [17, 18]. Therefore, TMS is a derivative of Res with a higher lipophilicity due to the introduction of three methyl groups, which improved the bioavailability to an absolute value of 45.4% [19]. The absorption rate constants of Res in the duodenum, jejunum, and ileum were 0.95, 0.52, and 0.38, respectively, and Res was distributed to every tissue and organ in the body at a nonlethal dose [20–22]. Three months after gavage at a dose of 120 mg/kg, no toxic reaction was observed in the liver, kidney, blood, and heart [23, 24].

The precise pathogenic mechanism underlying the development of PAH remains to be elucidated. The occurrence and development of PAH involve factors such as body fluids, cytokines, and genetics [25]. An increasing body of evidence points to the critical role of multiple cytokines involved in the inflammatory mechanism in the occurrence of PAH [26]. Currently, most people believe that the vital factors responsible for the occurrence and development of PAH include inflammatory responses involving cytokines, hypoxia, toxins, pathogens, and immune diseases that stimulate the release of inflammatory factors [27–30]. Among these inflammatory factors, NF-*κ*B, TNF-*α*, and IL-6 are the three most striking proinflammatory factors. The infiltration of large amounts of inflammatory cells, such as macrophages and lymphocytes, can be observed at pulmonary vascular sites in idiopathic PAH, which indicates that inflammatory factors play an important role in PAH.

NF-*κ*B is an important transcription factor that participates in the immune and inflammatory reactions that are responsible for the occurrence of PAH [31]. The transcriptional products of NF-*κ*B include cytokines, chemical factors, cell adhesion molecules, and immune receptors. Previous studies have shown that the increased activity of NF-*κ*B in the pulmonary artery endothelial cells of PAH rats inhibits the expression of vasoactive substances such as PGI_2_ [9]. Additionally, NF-*κ*B can also be activated by a low oxygen environment [32]. Myocardial apoptosis is inhibited by the activation of NF-*κ*B, which leads to myocardial proliferation and pulmonary vascular remodeling. One of the important components of pulmonary vascular remodeling is the increase in abnormal distribution of the extracellular matrix. The expression level of NF-*κ*B in myocardial cells, myocardial protein, and peripheral blood serum of the TMS-treated group was significantly lower than that of the model group. The synthesis and exocrine level of NF-*κ*B in myocardial tissue and peripheral blood can be inhibited by TMS, which, therefore, could play a role in treating and improving PAH.

TNF-*α* increases pulmonary vascular reactivity, reduces the synthesis of prostaglandins (PGs) in pulmonary artery smooth muscle cells, and induces pulmonary vasoconstriction [33]. COX is the key enzyme in the initial steps in the synthesis of PG. The level of COX-2 increases in accordance with the occurrence of PAH [34]. Nitric oxide synthase (NOS) is the key enzyme in the synthesis of NO [35]. Furthermore, inducible NOS (iNOS) is an important isoform of NOS that is expressed in small amounts under physiological conditions. Under pathological conditions such as hypoxia, the expression level is significantly increased, and excessive NO can combine with oxygen free radicals to generate peroxynitrite, which causes tissue damage [36].

The expression level of TNF-*α* in myocardial cells, myocardial protein, and peripheral blood serum in the TMS-treated group was significantly lower than that in the model group. Therefore, the synthesis and exocrine level of TNF-*α* in myocardial tissue and peripheral blood can be inhibited by TMS. These actions increase the synthesis of PG and inhibit the release of COX-2, thereby relieving pulmonary vasoconstriction and PAH symptoms.

IL-6 is a cytokine induced by IL-1 and TNF-*α* in mononuclear phagocytes [37]. Additionally, IL-6 is also an important target of Res and has multiple biological activities, including the activation of B and T cells and the production of KJ acute phase proteins [38]. IL-6 can be induced by IL-1*β* and TNF-*α* and by tissue damage [12]; moreover, IL-6 acts on B and T lymphocytes to affect the normal proliferation of myocardial cells by an autocrine effect. The synthesis of MMP increased IL-1 signaling, and the production of protein-polysaccharide is inhibited [39]. The expression levels of myocardial cells, myocardial protein, and peripheral blood serum in the TMS-treated group were significantly lower than those in the model group. The synthesis and exocrine level of IL-6 in myocardial tissue and peripheral blood can be inhibited by the TMS-induced inhibition of IL-1*β*, and therefore, TMS could have a potential role in treating and improving PAH.

In this study, we focused on trimethoxystilbene ameliorates pulmonary artery hypertension in rat model through reducing inflammation factors whereas the underlying molecular mechanisms were little involved, which is a limitation and worthy of a discussion. The possible mechanisms involved Akt-mediated Nrf2 transcription activation and p62-dependent Keap 1 degradation, by which attenuates the oxidative stress [40]. Resveratrol can activate the nuclear erythroid-2-like factor-2 (Nrf2) transcription factor. It is a master regulator of endogenous cellular defense mechanisms. Nrf2 controls the expression of many antioxidant and detoxification genes, by binding to antioxidant response elements (AREs) that are commonly found in the promoter region of antioxidant and other genes and that control the expression of those genes [41]. Resveratrol mediates the gene expression of vasoprotective factors that may counter the endothelial damage imposed by these antiangiogenic factors as well. Relative studies have revealed that Nrf2 knockdown abolished some of the protective effects of resveratrol on endothelial cells [42].

As mentioned above, the mechanism of the development of PAH is very complex. We treated the PAH SD rats with TMS and found that NF-*κ*B, IL-6, and TNF-*α* were inhibited in myocardial tissue and peripheral blood, which provides a potential new strategy and direction in exploring the possible mechanism of the development of PAH and the underlying mechanisms of TMS and other similar candidate treatments.

## Figures and Tables

**Table 1:** The comparison of ultrasound parameters in the control group and model group (*n* = 30).

Parameters	Control group	Model group	Standard deviation (SD)
RAWT (mm)	1.92 ± 0.19	2.5 ± 0.35	*P* < 0.05
RPWT (mm)	1.80 ± 0.1	2.8 ± 0.4	*P* < 0.05
RVSV (ml)	0.16 ± 0.04	0.13 ± 0.08	*P* < 0.05
EF (%)	84.4 ± 3.5	78.5 ± 5.9	*P* < 0.05
PAV (cm/s)	117 ± 18	149 ± 34	*P* < 0.05
HR (BPM)	467 ± 38	478 ± 48	

**Table 2:** Expression of nuclear factor kappa B (NF-*κ*B), interleukin- (IL-) 6, and tumor necrosis factor- (TNF-) *α* in myocardial cells from pulmonary artery hypertension (PAH) model rats.

Group	n	Positive cell rate (%)		
		NF-*κ*B	IL-6	TNF-*α*
Normal	12	2.912 ± 0.903	2.152 ± 0.765	3.186 ± 1.332
Model	12	49.067 ± 5.545^*∗*^	27.148 ± 4.132^*∗*^	37.892 ± 11.432^*∗*^
TMS	12	11.821 ± 3.114^*∗∗*^	5.826 ± 1.772^*∗∗*^	9.381 ± 2.329^*∗∗*^

^*∗*^ *P* < 0.01, model group vs normal control group comparison. ^*∗∗*^*P* < 0.01, trimethoxystilbene (TMS) group vs model group comparison.

**Table 3:** Expression and grayscale scanning values of nuclear factor kappa B (NF-*κ*b), interleukin- (IL-) 6, and tumor necrosis factor- (TNF-) *α* in the myocardial cells of pulmonary artery hypertension (PAH) model SD rats as determined using western blotting.

Group	NF-*κ*B	IL-6	TNF-*α*	*β*-actin
TMS	3029.62 ± 1172.29^*∗∗*^	4482.32 ± 1972.83^*∗∗*^	3789.21 ± 1092.14^*∗∗*^	8128.29 ± 2891.84
PAH	7573.43 ± 2374.32^*∗*^	3773.82 ± 1332.44^*∗*^	5586.36 ± 1886.92^*∗*^	7846.82 ± 2602.48
Normal	2294.41 ± 1248.32	2184.04 ± 1208.58	1989.37 ± 1189.29	8032.53 ± 2788.21
TMS/*β*-actin	0.373 ± 0.094	0.551 ± 0.126	0.466 ± 0.113	
PAH/*β*-actin	0.965 ± 0.138	0.430 ± 0.117	0.712 ± 0.152	
Normal/*β*-actin	0.286 ± 0.083	0.272 ± 0.088	0.247 ± 0.062	

^*∗*^ *P* < 0.01, model group vs normal control group comparison. ^*∗∗*^*P* < 0.01, trimethoxystilbene (TMS) group vs model group comparison.

**Table 4:** Expression level of interleukin- (IL-) 6 and tumor necrosis factor- (TNF-) *α* in the serum of pulmonary arterial hypertension (PAH) model rats as determined using enzyme-linked immunosorbent assay (ELISA).

Group (*n* = 12)	IL-6 (ng/L)	TNF-*α* (ng/L)
TMS	11.32 ± 3.75^*∗∗*^	16.82 ± 4.48^*∗∗*^
PAH	37.41 ± 5.97^*∗*^	46.31 ± 6.31^*∗*^
Normal	7.64 ± 1.00	11.35 ± 0.55

^*∗*^ *P* < 0.01, model group vs normal control group comparison. ^*∗∗*^*P* < 0.01, trimethoxystilbene (TMS) group vs model group comparison.

## Data Availability

The experimental data used to support the findings of this study are included in the article.
