# Erratum to “Trimethoxystilbene Reduces Nuclear Factor Kappa B, Interleukin-6, and Tumor Necrosis Factor-*α* Levels in Rats with Pulmonary Artery Hypertension”

**DOI:** 10.1155/2020/1892431

**Published:** 2020-09-10

**Authors:** Jie Shu, Wei Liu, Fei Han, Fanyan Luo

**Affiliations:** The Department of Cardiothoracic Surgery, Xiangya Hospital, Central South University, Changsha, Hunan Province, China

In the article titled “Trimethoxystilbene Reduces Nuclear Factor Kappa B, Interleukin-6, and Tumor Necrosis Factor-*α* Levels in Rats with Pulmonary Artery Hypertension” [1], information was omitted in the Authors' Contributions section. The corrected section appears below:
